# Allele and Haplotype Diversity of 26 X-STR Loci in Four Nationality Populations from China

**DOI:** 10.1371/journal.pone.0065570

**Published:** 2013-06-21

**Authors:** Qiu-Ling Liu, Jing-Zhou Wang, Li Quan, Hu Zhao, Ye-Da Wu, Xiao-Ling Huang, De-Jian Lu

**Affiliations:** 1 Faculty of Forensic Medicine, Zhongshan School of Medicine, Sun Yat-sen University, Guangzhou, P.R. China; 2 Inner Mongolia Public Security Departments, the Criminal Investigation Division, Inner Mongolia, P.R. China; 3 Shanghai Key Laboratory of Forensic Medicine, Institute of Forensic Sciences, Ministry of Justice, Shanghai, P.R China; St. Petersburg Pasteur Institute, Russian Federation

## Abstract

**Background:**

Haplotype analysis of closely associated markers has proven to be a powerful tool in kinship analysis, especially when short tandem repeats (STR) fail to resolve uncertainty in relationship analysis. STR located on the X chromosome show stronger linkage disequilibrium compared with autosomal STR. So, it is necessary to estimate the haplotype frequencies directly from population studies as linkage disequilibrium is population-specific.

**Methodology and Findings:**

Twenty-six X-STR loci including six clusters of linked markers DXS6807-DXS8378-DXS9902(Xp22), DXS7132-DXS10079-DXS10074-DXS10075-DXS981 (Xq12), DXS6801-DXS6809-DXS6789-DXS6799(Xq21), DXS7424-DXS101-DXS7133(Xq22), DXS6804-GATA172D05(Xq23), DXS8377-DXS7423 (Xq28) and the loci DXS6800, DXS6803, DXS9898, GATA165B12, DXS6854, HPRTB and GATA31E08 were typed in four nationality (Han, Uigur, Kazakh and Mongol) samples from China (n = 1522, 876 males and 646 females). Allele and haplotype frequency as well as linkage disequilibrium data for kinship calculation were observed. The allele frequency distribution among different populations was compared. A total of 5–20 alleles for each locus were observed and altogether 289 alleles for all the selected loci were found. Allele frequency distribution for most X-STR loci is different in different populations. A total of 876 male samples were investigated by haplotype analysis and for linkage disequilibrium. A total of 89, 703, 335, 147, 39 and 63 haplotypes were observed. Haplotype diversity was 0.9584, 0.9994, 0.9935, 0.9736, 0.9427 and 0.9571 for cluster I, II, III, IV, V and VI, respectively. Eighty-two percent of the haplotype of cluster IIwas found only once. And 94% of the haplotype of cluster III show a frequency of <1%.

**Conclusions:**

These results indicate that allele frequency distribution for most X-STR loci is population-specific and haplotypes of six clusters provide a powerful tool for kinship testing and relationship investigation. So it is necessary to obtain allele frequency and haplotypes data of the linked loci for forensic application.

## Introduction

**Figure 1:**
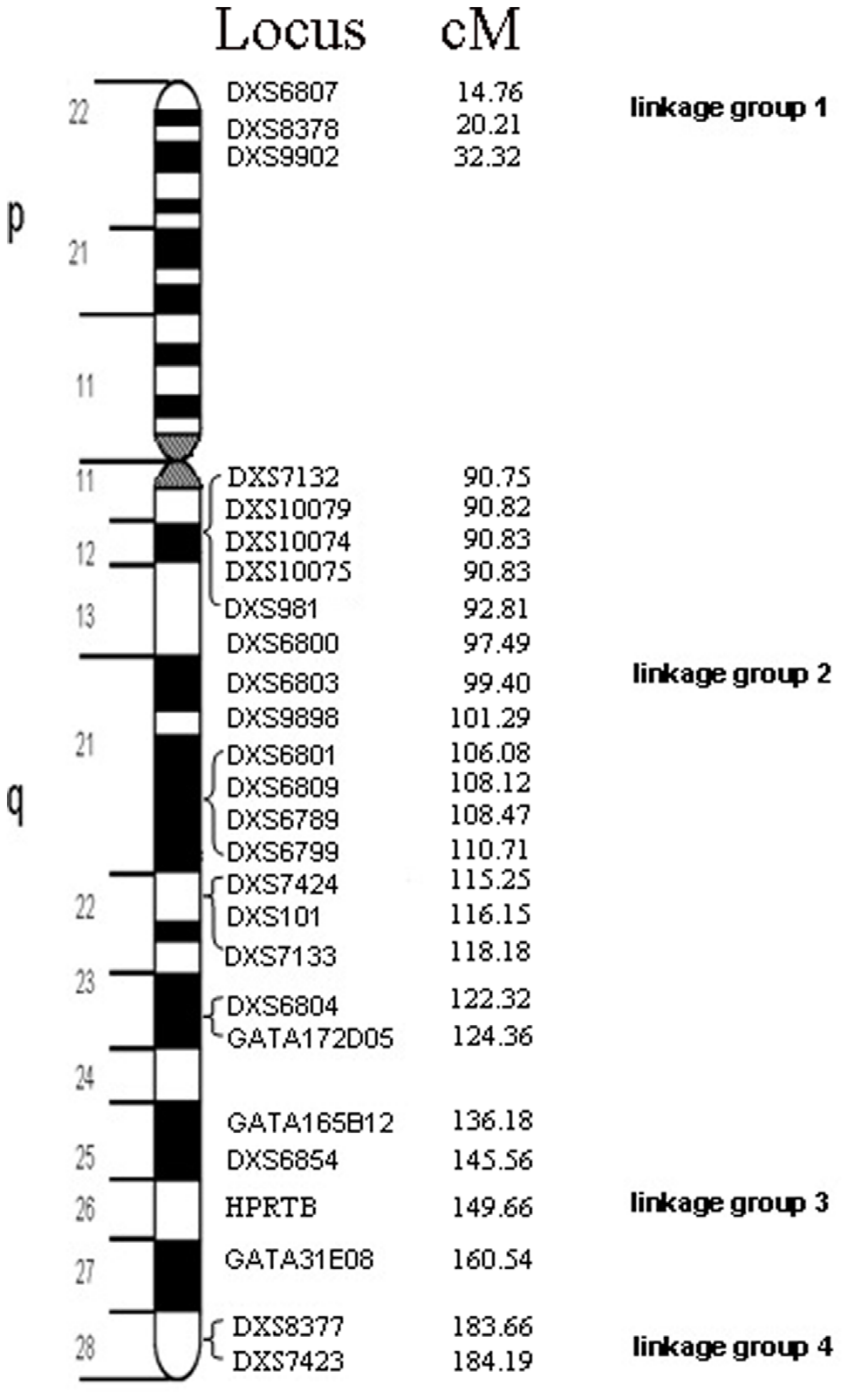
Idiogram of 26 X-STR Loci.

Autosomal short tandem repeats (AS-STR) and Y chromosomal STR (Y-STR) are powerful tools for human identification and kinship test. Many multiplex PCR systems of autosomal STR (AS-STR) and Y chromosomal STR (Y-STR) have been reported, and many commercial kits of the AS-STR and the Y-STR are available. The X chromosomal STR (X-STR) is recognized as important tools in forensic application. In recent years, considerable X-STR systems have been studied in the field of population genetics and forensics [1]–[5]. However, few kits include X-linked X-STR markers except Mentype® Argus X-8 Kit and Investigator Argus X-12 Kit (Biotype AG, Dresden, Germany). With the complication of forensic cases, AS-STR and the Y-STR markers as well as these two X-STR Kits were not enough in forensic application. So we developed two multiplex PCR system with twenty-six X-STR loci including DXS6800(Xq13), DXS6803(Xq21), DXS9898(Xq21), GATA165B12 (Xq25), DXS6854(Xq25), HPRTB(Xq26), GATA31E08 (Xq27), and six clusters of closely linked markers, cluster I: DXS6807-DXS8378-DXS9902 (Xp22); II: DXS7132-DXS10079-DXS10074-DXS10075-DXS981 (Xq12); III: DXS6801-DXS6809- DXS6789-DXS6799 (Xq21); IV: DXS7424-DXS101-DXS7133 (Xq22); V: DXS6804- GATA172D05 (Xq23); and VI: DXS8377-DXS7423 (Xq28). (Fig. 1 shows the physical localization of these markers). On the other hand, allele frequency distribution for most X-STR loci varies with different populations [6], [7]. Moreover, the use of X-STR requires a precise knowledge not only of allele and haplotype frequencies, but also of the genetic linkage and linkage disequilibrium (LDE) status among markers [8]. This study investigated polymorphism and linkage and/or independence of the selected markers in four nationality populations from China.

## Materials and Methods

### Sampling and DNA extraction

Blood samples were collected from 1,522 unrelated individuals from four nationality populations in Mainland China. A total of 745 subjects of Han nationality from Guangdong (477 males and 268 females), 234 subjects of Uigur nationality (100 males and 134 females) from Yi-ning City, Ili, Xinjiang Province, 386 subjects of Kazakh nationality (173 males and 213 females) from Tacheng Prefecture of Xinjiang and 157 subjects of Mongol nationality (126 males and 31 females) from Inner Mongolia were studied. There were 325 family trios (father-mother-daughter), 286 family duos (mother-son), and 40 three-generation families (grandmother-father-granddaughter) from Guangdong. Parents of the trios and mothers of the duos were included in the unrelated individuals. Samples were prepared and DNA was extracted using Chelex-100 methods [9].

### Ethics Statement

The research protocol was approved by the Human Subjects Committee at the Zhongshan School of Medicine, Sun Yat-sen University and written informed consent was obtained from all participants or guardians involved in the study.

### PCR amplification

All of samples were genotyped for 26 X-STR loci in two multiplex systems including MX15-STR and MX12-STR. MX15-STR consisted of DXS7133, DXS6801, DXS981, DXS6809, DXS7424, DXS6789, DXS9898, DXS7132, GATA165B12, DXS101, DXS10075, DXS6800, GATA31E08, DXS10074 and DXS10079 in a single multiplex reaction, in which primer and PCR conditions were as described elsewhere [10]. MX12-STR consisted of DXS6854, DXS9902, DXS6800, GATA172D05, DXS7423, HPRTB, DXS6807, DXS6803, DXS6804, DXS6799, DXS8378 and DXS8377 in a single multiplex reaction, in which primer and PCR conditions were as described elsewhere [11].

### Sample electrophoresis

Electrophoresis was performed in a 24-capillary ABI 3500 Genetic Analyzer (Applied Biosystems, USA). 1 µl PCR products to 10 µl deionized formamide (Applied Biosystems, USA) and 0.25 µl Genescan™-500 LIZ™ size standards (Applied Biosystems, USA). The matrix standards for spectral calibration were developed according to the Matrix manufacture's instructions (AGCU Scien Tech Incorporation, China). The results were analyzed with GeneMapper ID-X Analysis Software. The K562 and 9947A (Promega Corporation, Madison, WI, USA) Cell lines DNA were typed for calibrating allelic ladder.

### Sequence analysis

Allele of the ladder was sequenced in order to ensure correct designation of allele nomenclature. Samples were amplified with the single PCR in Gene Amp PCR System 9700 Thermal Cycler (Applied Biosystems, Foster City, CA, USA) under the following conditions: initial denaturation at 94°C for 11 min, followed by 30 cycles of 94°C for 45 min, 61°C for 45 min, 72°C for 45 min, and additional 72 min at 5°C. PCR products were purified or cloned with the TOP10F Cloning Kit (TIANGEN Biochemical Technology Co. Beijing, China) following the manufacturer's instructions. Then purified PCR products or the chosen clones were sequenced on ABI 3100 Genetic Analyzer using a BigDye® Terminator Cycle Sequencing Kit (Applied Biosystems, USA) according to the manufacturer's instructions.

### Statistical analysis

The software ARLEQUIN 3.5 [12] was used to perform the following statistical analysis, including allelic frequencies and haplotype frequencies, the exact chi-square test for Hardy-Weinberg equilibrium (HWE) for female data, exact tests for population differentiation between allele frequencies of males and females, linkage disequilibrium (LDE) test between all pairs of markers. The exact test differentiation of allele frequency distribution among different populations was performed with SPSS v.15.0. Polymorphism information content (PIC) was estimated according to Botstein et al. [13] The power of discrimination in females (PD_F_) and males (PD_M_), mean exclusion chance (MEC) were calculated according to Desmarais et al. [14]

## Results

Sequences of some alleles for ladder are shown in electronic supplementary material (ESM: FigS1, FigS2, FigS3, FigS4, FigS5, FigS6, FigS7, FigS8, FigS9, FigS10, FigS11, FigS12, FigS13, FigS14, FigS15, FigS16, FigS17, FigS18, FigS19, FigS20, FigS21, FigS22, FigS23, FigS24, FigS25, FigS26, FigS27, FigS28 in File S1). When 1,522 samples were tested, a total of 5–20 alleles for each locus were observed and altogether 289 alleles for all the selected loci were found. The allele frequencies and further statistical information of the twenty-six loci in Han, Uigur and Mongol population are shown in Table 1. The allele frequencies and further statistical information in Kazakh has been described in MX15-STR [10] and MX12-STR [11]. HWE was performed on female samples, and the P-values of HWE are greater than 0.05 at all the twenty-six loci. The comparisons among our studied populations as well as between our selected populations and those reported by others show that allele frequency distribution is different for most X-STR loci in different populations. The results for P-values of population differentiation are listed in Table S1 and Table S2. A total of 876 male samples were investigated by haplotype analysis and for linkage disequilibrium. *P* valuate of the exact test for LDE is listed in Table 2. The haplotype number and haplotype diversity of the six clusters are shown Table 3. The haplotype frequencies of the six clusters are shown in Table S3, S4, S5, S6, S7, and S8. Thirty-one cases of mutation were detected from the fifteen loci in 9,480 meioses. Mutation information is listed in Table 4.

**Table 1:** Allele frequencies and statistical parameter of the 26 loci in the three nationality populations from China.

Allele	DXS7133			GATA165B12			Allele	GATA31E08			Allele	DXS6801		
	Han	Uigur	Mongol	Han	Uigur	Mongol		Han	Uigur	Mongol		Han	Uigur	Mongol
6		0.0054	0.0053				5		0.0027		8	0.0010		
7	0.0010	0.0027		0.0020	0.0054		6		0.0027		9		0.0027	0.0106
8	0.0010	0.0027	0.0053	0.0020			7	0.1244	0.1359	0.0957	10	0.1323	0.1440	0.2021
9	0.7907	0.5897	0.6277	0.2922	0.2717	0.1968	8	0.0306	0.0326	0.0426	11	0.5814	0.5679	0.5532
10	0.1550	0.1929	0.2500	0.5192	0.5027	0.5638	9	0.2251	0.2446	0.2553	12	0.2093	0.1957	0.1649
11	0.0503	0.1848	0.1064	0.1550	0.1984	0.2074	10	0.2488	0.2310	0.1862	13	0.0701	0.0734	0.0638
12	0.0020	0.0191	0.0053	0.0296	0.0163	0.0319	11	0.2794	0.2772	0.3085	14	0.0049	0.0136	0.0053
13		0.0027			0.0054		12	0.0770	0.0707	0.0957	15	0.0010	0.0027	
							13	0.0138	0.0027	0.0160				
							14	0.0010						
K562	10			10				11				11		
9947A	9,10			9,11				11				11		
*PD_M_*	0.3442	0.5890	0.5249	0.6183	0.6510	0.5897		0.7929	0.7910	0.7773		0.5719	0.6274	0.6064
*PD_F_*	0.5441	0.7677	0.7414	0.7904	0.7953	0.7875		0.9181	0.9167	0.9249		0.8017	0.8027	0.8217
*MECI*	0.3149	0.5280	0.4725	0.5563	0.5705	0.5432		0.7542	0.7521	0.7528		0.5488	0.5689	0.5747
*MECII*	0.1946	0.3795	0.3284	0.4089	0.4224	0.3944		0.6248	0.6221	0.6234		0.3998	0.4198	0.4257
*PIC*	0.3482	0.5805	0.5321	0.6201	0.6338	0.5993		0.7869	0.7855	0.7846		0.5957	0.6128	0.6217

Allele	DXS6799			DXS6804			Allele	DXS6807			Allele	HPRTB		
	Han	Uigur	Mongol	Han	Uigur	Mongol		Han	Uigur	Mongol		Han	Uigur	Mongol
8	0.0069	0.0136	0.0160	0.0039	0.0027		9	0.0020			10	0.0030		
9	0.0168	0.0435	0.0426			0.0053	11	0.4798	0.4620	0.5053	11	0.0800	0.0679	0.0426
10	0.1343	0.2636	0.1543				12	0.0207	0.0462	0.0266	12	0.2794	0.2853	0.2074
11	0.6387	0.4701	0.5957	0.2241	0.2228	0.2766	13	0.0178	0.0380	0.0691	13	0.4393	0.3750	0.3936
12	0.1639	0.1603	0.1489	0.1540	0.1495	0.1277	14	0.3159	0.2663	0.2340	13.2	0.0010		
13	0.0375	0.0435	0.0319	0.3475	0.4212	0.3085	15	0.1461	0.1603	0.1330	14	0.1422	0.1957	0.2872
14	0.0020	0.0054	0.0106	0.1994	0.1495	0.1596	16	0.0168	0.0217	0.0319	15	0.0474	0.0707	0.0691
15				0.0681	0.0543	0.1117	17	0.0010	0.0054		16	0.0069	0.0054	
16				0.0020		0.0106					17	0.0010		
17				0.0010										
K562	11			14				11				13		
9947A	11,12			13,15				12,14				14		
*PD_M_*	0.5481	0.5728	0.5999	0.7525	0.6942	0.7739		0.6423	0.6626	0.6612		0.7100	0.7270	0.7081
*PD_F_*	0.7507	0.8072	0.7822	0.9088	0.8882	0.9116		0.8186	0.8608	0.8530		0.8533	0.8735	0.8764
*MECI*	0.5063	0.6316	0.5597	0.7230	0.6848	0.7388		0.5869	0.6385	0.6225		0.6539	0.6851	0.6632
*MECII*	0.3583	0.4861	0.4104	0.5869	0.5432	0.6059		0.4404	0.4935	0.4760		0.5102	0.5442	0.5199
*PIC*	0.5454	0.6798	0.5959	0.7609	0.7253	0.7739		0.6477	0.6859	0.6657		0.7001	0.7300	0.7129

Allele	DXS6854			DXS8378				DXS9902				GATA172D05		
	Han	Uigur	Mongol	Han	Uigur	Mongol		Han	Uigur	Mongol		Han	Uigur	Mongol
6	0.0010											0.0405	0.106	0.0532
7	0.0010	0.0027						0.0020	0.0109			0.0099	0.0054	0.0106
8		0.0054						0.0010				0.2053	0.1060	0.1011
9	0.0415	0.1413		0.0257	0.0326	0.0106		0.0138	0.0435	0.0160		0.1224	0.0543	0.0426
10	0.2843	0.2908	0.1755	0.5163	0.4076	0.5319		0.4600	0.3397	0.4468		0.3672	0.3995	0.4521
11	0.3712	0.0870	0.4362	0.2981	0.3424	0.2713		0.3297	0.3723	0.3351		0.2113	0.2500	0.2819
12	0.1273	0.2826	0.1064	0.1422	0.1712	0.1702		0.1826	0.2147	0.1968		0.0434	0.0788	0.0585
13	0.1254	0.1576	0.1968	0.0118	0.0408	0.0053		0.0099	0.0190	0.0053				
14	0.0415	0.0299	0.0691	0.0059	0.0054	0.0106								
15	0.0059	0.0027	0.0106					0.001						
16	0.0010													
17			0.0053											
K562	14			10				11,12				12		
9947A	13,14			10,11				11				10		
*PD_M_*	0.7389	0.7784	0.7531	0.6164	0.6792	0.5863		0.6391	0.668	0.644		0.7702	0.7678	0.7129
*PD_F_*	0.9011	0.9206	0.8279	0.8018	0.8396	0.8113		0.8121	0.8574	0.7903		0.8997	0.8941	0.8161
*MECI*	0.7075	0.7495	0.6866	0.5612	0.6273	0.5517		0.5784	0.6408	0.5798		0.7242	0.7116	0.6546
*MECII*	0.5703	0.6191	0.5455	0.4141	0.4821	0.4039		0.4313	0.4959	0.4326		0.5891	0.575	0.5117
*PIC*	0.746	0.7823	0.724	0.6235	0.6846	0.6142		0.646	0.6976	0.649		0.7598	0.7463	0.6977

Allele	DXS6789			DXS6800			Allele	DXS10079			Allele	DXS101		
	Han	Uigur	Mongol	Han	Uigur	Mongol		Han	Uigur	Mongol		Han	Uigur	Mongol
14	0.0039	0.0082					14			0.0053	17		0.0054	
15	0.1688	0.0870	0.1170	0.0020	0.0027		15	0.0049	0.0109	0.0106	18		0.0191	0.0053
16	0.3406	0.1630	0.2340	0.8164	0.6277	0.8085	16	0.0138	0.0435	0.0372	19	0.0109	0.0163	
17	0.0533	0.0027	0.0213	0.0030	0.0299	0.0053	17	0.0642	0.0679	0.0745	20	0.0020	0.0109	0.0160
18	0.0010			0.0178	0.0815	0.0213	18	0.1244	0.1223	0.1436	21	0.0128	0.0190	0.0053
19	0.0178	0.0571	0.0798	0.0908	0.1603	0.1064	18.2	0.2468	0.0027		22	0.0661	0.0380	0.0532
20	0.2122	0.3397	0.2553	0.0257	0.0054	0.0106	19	0.2695	0.2690	0.1809	23	0.0997	0.0707	0.1223
21	0.1451	0.2011	0.1968	0.0217	0.0571	0.0426	20	0.1530	0.2554	0.1968	24	0.2853	0.2473	0.2500
22	0.0494	0.0897	0.0851	0.0227	0.0353	0.0053	21	0.0908	0.1304	0.1862	25	0.2053	0.1984	0.2128
23	0.0079	0.0408	0.0106				22	0.0267	0.0842	0.1436	26	0.1708	0.1821	0.2394
24		0.0109					23	0.0039	0.0109	0.0213	27	0.0967	0.1005	0.0532
25							24		0.0027		28	0.0316	0.0489	0.0372
26							25	0.0020			29	0.0168	0.0272	
27											30	0.0020	0.0054	0.0053
28											31		0.0054	
29											32		0.0027	
											33		0.0027	
K562	21			21				17				24		
9947A	21,22			18,19				20,23				24,26		
*PD_M_*	0.7764	0.8148	0.7943	0.4270	0.5922	0.2954		0.8045	0.8444	0.8403		0.8222	0.8612	0.8147
*PD_F_*	0.9264	0.9279	0.9529	0.3827	0.7734	0.6122		0.9441	0.9351	0.9568		0.9452	0.9530	0.9352
*MECI*	0.7537	0.7714	0.7877	0.3096	0.5377	0.3145		0.7896	0.7930	0.8258		0.7998	0.8281	0.7869
*MECII*	0.6247	0.6471	0.6667	0.1915	0.3886	0.1949		0.6697	0.6743	0.7166		0.6832	0.7215	0.6663
*PIC*	0.7837	0.7969	0.8134	0.3233	0.5682	0.3326		0.8143	0.8166	0.8451		0.8220	0.8455	0.8126

Allele	DXS7424			DXS7423			Allele	DXS9898			Allele	DXS7132		
	Han	Uigur	Mongol	Han	Uigur	Mongol		Han	Uigur	Mongol		Han	Uigur	Mongol
10.2				0.0010			8.3	0.0296	0.1440	0.0585	9			0.0053
11	0.0079	0.0054	0.0106	0.0010			9	0.0020			10			
12	0.0039	0.0163	0.0053				10	0.0010	0.0136	0.0053	11	0.0049	0.0082	0.0106
13	0.0197	0.0272	0.0585	0.0030	0.0299	0.0106	11	0.1007	0.0897	0.1117	12	0.0888	0.0978	0.0479
14	0.1264	0.1359	0.1436	0.3771	0.3016	0.2500	12	0.5143	0.4429	0.4255	13	0.1935	0.2364	0.2340
15	0.3564	0.2799	0.3191	0.5805	0.5272	0.6277	13	0.2606	0.2038	0.3191	14	0.3583	0.3342	0.3723
16	0.3801	0.3723	0.3457	0.0365	0.1223	0.0904	14	0.0839	0.0870	0.0745	15	0.2596	0.2663	0.2553
17	0.0760	0.1359	0.0904	0.0010	0.0190	0.0160	15	0.0079	0.0163	0.0053	16	0.0800	0.0516	0.0585
18	0.0237	0.0136	0.0266			0.0053	16		0.0027		17	0.0138	0.0054	0.0160
19	0.0010										18	0.0010		
20	0.0010	0.0082												
21	0.0039	0.0054												
K562	17			17				12				13		
9947A	14,16			14,15				12,15				12		
*PD_M_*	0.7059	0.7370	0.7443	0.6359	0.7306	0.6668		0.7508	0.7290	0.7333		0.5189	0.5864	0.4759
*PD_F_*	0.8636	0.8944	0.8971	0.8372	0.8857	0.8915		0.9003	0.8980	0.8861		0.6728	0.8004	0.7796
*MECI*	0.6565	0.7057	0.7059	0.6013	0.6909	0.6462		0.7134	0.7069	0.6917		0.4224	0.5523	0.4779
*MECII*	0.5142	0.5682	0.5688	0.4543	0.5507	0.5027		0.5765	0.5688	0.5520		0.2883	0.4056	0.3336
*PIC*	0.7058	0.7448	0.7455	0.6494	0.7255	0.6956		0.7522	0.7491	0.7353		0.5195	0.6149	0.5350

Allele	DXS6809						Allele	DXS8377			Allele	DXS10075		
	Han	Uigur	Mongol					Han	Uigur	Mongol		Han	Uigur	Mongol
27			0.0106				41	0.0039	0.0027		13			0.0053
28	0.0030	0.0027					42	0.0039	0.0136	0.0053	13.2		0.0054	
29	0.0109	0.0109	0.0160				43	0.0188	0.0217	0.0213	14		0.0109	
30	0.0375	0.0543	0.0160				44	0.0267	0.0489	0.0479	14.2		0.0136	0.0053
31	0.1422	0.1712	0.1277				45	0.0602	0.0761	0.0851	15	0.0138	0.0109	0.0160
32	0.1619	0.1685	0.1596				46	0.1066	0.1114	0.1223	15.2		0.0054	
33	0.2290	0.3261	0.3245				47	0.1106	0.0897	0.1117	16	0.2093	0.2364	0.1436
34	0.2320	0.1766	0.2128				48	0.1382	0.1168	0.0851	16.2	0.0276	0.0245	0.0372
35	0.1125	0.0598	0.1064				49	0.1076	0.1359	0.1383	16.3			0.0106
36	0.0602	0.0245	0.0266				50	0.1135	0.0978	0.1117	17	0.4551	0.4239	0.4255
37	0.0089						51	0.1185	0.1005	0.1117	17.1			0.0053
38	0.0020	0.0027					52	0.0523	0.0625	0.0957	17.2	0.0227	0.0190	0.0372
39		0.0027					53	0.0503	0.0435	0.0266	18	0.2488	0.2283	0.2606
							54	0.0503	0.0435	0.0213	18.2	0.0020		0.0106
							55	0.0168	0.0082	0.0106	19	0.0158	0.0082	0.0372
							56	0.0069	0.0163	0.0053	20	0.0020	0.0136	0.0053
							57	0.0059	0.0027		22	0.0030		
							58	0.0039	0.0082					
							59	0.0049						
K562	34						52				18			
9947A	31,34						45,47				16,19			
*PD_M_*	0.8283	0.7906	0.7962					0.8990	0.9054	0.9015		0.6876	0.7066	0.7048
*PD_F_*	0.9490	0.9321	0.9166					0.9849	0.9847	0.9758		0.8465	0.8669	0.9100
*MECI*	0.8072	0.7705	0.7672					0.8982	0.9039	0.8921		0.6352	0.6650	0.6882
*MECII*	0.6921	0.6454	0.6413					0.8220	0.8308	0.8124		0.4898	0.5225	0.5491
*PIC*	0.8294	0.7975	0.7950					0.9059	0.9108	0.9007		0.6855	0.7106	0.7256

Allele	DXS6803						Allele	DXS981			Allele	DXS10074		
	Han	Uigur	Mongol					Han	Uigur	Mongol		Han	Uigur	Mongol
8		0.0027					11	0.0010	0.0082		7.3			0.0053
9	0.0059	0.0027	0.0213				11.3	0.0010	0.0027	0.0053	8		0.0163	
9.3	0.0030	0.0245					12	0.0444	0.0842	0.0213	8.2			0.0053
10	0.1481	0.1875	0.1596				12.3	0.0592	0.0027	0.0638	9		0.0353	
10.3	0.0316	0.0870	0.1383				13	0.1658	0.1359	0.1383	10		0.0027	
11	0.1343	0.1793	0.1702				13.3	0.1945	0.2310	0.1330	11	0.0030	0.0027	
11.3	0.3337	0.3043	0.3936				14	0.2507	0.2092	0.3085	13	0.0039	0.0082	
12	0.2172	0.0543	0.0160				14.3	0.0948	0.1304	0.1489	14	0.0188	0.0245	0.0053
12.3	0.0721	0.1440	0.0904				15	0.1412	0.1277	0.1223	14.2		0.0027	
13	0.0405	0.0082					15.3	0.0128	0.0190	0.0319	15	0.0652	0.0652	0.0213
13.3	0.0059	0.0054	0.0106				16	0.0276	0.0353	0.0160	15.3			0.0053
14	0.0059						16.3	0.0010	0.0054	0.0106	16	0.1807	0.2418	0.2287
14.3	0.0020						17	0.0030	0.0082		17	0.3455	0.2880	0.3830
							17.3	0.0020			18	0.2369	0.2092	0.2074
							18	0.0010			19	0.1135	0.0842	0.1170
											20	0.0296	0.0192	0.0213
											21	0.0030		
K562	9						13.3				17			
9947A	10.3,11						13.3,14.3				16,19			
*PD_M_*	0.7891	0.7940	0.7423					0.8314	0.8268	0.8254		0.7729	0.7958	0.7640
*PD_F_*	0.9324	0.9392	0.9305					0.9531	0.9561	0.9461		0.9155	0.9317	0.8544
*MECI*	0.7665	0.7826	0.7320					0.8164	0.8230	0.8047		0.7408	0.7735	0.7036
*MECII*	0.6407	0.6605	0.5984					0.7046	0.7133	0.6896		0.6091	0.6498	0.5652
*PIC*	0.7936	0.8081	0.7625					0.8365	0.8422	0.8249		0.7735	0.8008	0.7433

*PD_M_* power of discrimination in males, *PD_F_* power of discrimination in females, *MEC I* mean exclusion chance for X-STR in standard trios with daughters. *MEC II* mean exclusion chance for X-STR in father/daughter duos. *PIC:* polymorphism information content.

**Table 2:** Results of *p* values for test of linkage disequilibrium.

Locus by locus	Han	Uigur	Kazakh	Mongol
Cluster I				
DXS6807-DXS8378	0.0602	0.2132	0.7077	0.5559
DXS6807-DXS9902	0.0941	0.5605	0.4133	0.6193
DXS8378-DXS9902	0.0051	0.0427	0.9381	0.3031
Cluster II				
DXS7132-DXS10079	0.5232	0.2872	0.0144	0.8170
DXS7132-DXS10074	0.3411	**0.0013**	0.1079	0.8794
DXS10079-DXS10074	0.8413	0.0181	0.0866	0.8582
DXS7132-DXS10075	0.6370	0.5349	0.7980	0.3982
DXS10079-DXS10075	**0.0000**	**0.0000**	0.3595	0.3246
DXS10074-DXS10075	0.0857	0.1773	0.0671	0.0582
DXS7132-DXS981	0.2307	0.4397	0.1836	0.5465
DXS10079-DXS981	0.4329	0.2316	0.2283	0.9037
DXS10074-DXS981	0.1102	0.5168	0.2854	0.8971
DXS10075-DXS981	0.0962	**0.0072**	0.3877	0.1174
Cluster III				
DXS6801-DXS6809	0.7288	0.0228	0.5766	0.3312
DXS6801-DXS6789	0.4283	**0.0000**	0.4185	**0.0126**
DXS6809-DXS6789	0.0855	**0.0000**	0.2871	**0.0498**
DXS6801-DXS6799	0.6296	0.9154	0.2324	0.2451
DXS6809-DXS6799	0.3108	0.8321	0.2323	0.5647
DXS6789-DXS6799	0.4765	0.6542	0.6777	0.1930
Cluster IV				
DXS7424-DXS101	0.1179	0.0555	0.0124	0.1493
DXS7424-DXS7133	0.0428	0.0049	0.0000	0.0186
DXS101-DXS7133	0.9762	0.3551	0.0432	0.9536
Cluster V				
DXS6804-GATA172D05	0.0078	0.0096	0.2969	0.1108
Cluster VI				
DXS8377-DXS7423	0.0473	0.0523	0.5759	0.4960

**Table 3:** Haplotype number and diversity of the six clusters in the four nationality populations from China.

Sample number Clusters	Haplotype number					Haplotype diversity				
	Han 477	Uigur 100	Kazakh 173	Mongol 126	Total 876	Han 477	Uigur 100	Kazakh 173	Mongol 126	Total 876
I: DXS6807/DXS8378/DXS9902	66	36	57	37	89	0.9505	0.9657	0.9706	0.9581	0.9584
II: DXS7132/DXS10079/DXS10074/DXS10075/DXS981	404	86	166	121	703	0.9991	0.9971	0.9996	0.9994	0.9994
III: DXS6801/DXS6809/DXS6789/DXS6799	222	73	112	90	335	0.9922	0.9921	0.9921	0.9914	0.9935
IV: DXS7424/DXS101/DXS7133	96	56	46	35	147	0.9651	0.9817	0.9807	0.9774	0.9736
V: DXS6804/GATA172D05	34	24	31	31	39	0.9417	0.9239	0.9420	0.9346	0.9427
VI: DXS8377/DXS7423	45	33	46	35	63	0.9514	0.9623	0.9641	0.9524	0.9571

**Table 4:** Mutation detected from the pedigree analysis of the 325 father-daughter-mother trios and the 286 mother-son duos.

Locus	Genotype			Transmission	Age	Mutation rate(%)
	Father	Mother	Child*			
DXS9902	12	10-10	**11–12**	Mother to Daughter	Father(35); Mother(23)	0.0011
DXS7132	14	13–14	13–**15**	Father to Daughter	Father(28); Mother(30)	0.0032
DXS7132	15	12–15	12–14	Father to Daughter	Father(40); Mother(30)	
DXS7132		14–17	**13**	Mother to Son	Mother(25)	
DXS10079	20	19–22	19-**19**	Father to Daughter	Father(24); Mother(22)	0.0043
DXS10079	20	17–21	20-**20**	Mother to Daughter	Father(35); Mother(30)	
DXS10079	20	18–19	18–**19**	Father to Daughter	Father(30); Mother(28)	
DXS10079	18	22-22	**19–**22	Father to Daughter	Father(26); Mother(22)	
DXS10074	20	16–17	16–**19**	Father to Daughter	Father(22); Mother(21)	0.0021
DXS10074		17-17	**18**	Mother to Son	Mother(22)	
DXS10075	18	16–18	16–**19**	Father to Daughter	Father(33); Mother(31)	0.0043
DXS10075	18	17-17	18-**18**	Mother to Daughter	Father(38); Mother(31)	
DXS10075	17	16–17	17–**18**	uncertain	Father(30); Mother(29)	
DXS10075	18	17–18	18–**19**	uncertain	Father(26); Mother(20)	
DXS6803	10	10–11.3	**11–**11.3	Father to Daughter	Father(36); Mother(34)	0.0011
DXS6809	32	31–36	31–**33**	Father to Daughter	Father(32); Mother(24)	0.0021
DXS6809	34	30–34	30–**35**	Father to Daughter	Father(35); Mother(25)	
DXS6789	16	20–21	**17–**20	Father to Daughter	Father(2); Mother(25)	0.0011
DXS7424	16	11–15	16-**16**	Mother to Daughter	Father(29); Mother(24)	0.0043
DXS7424	16	15-15	**14–**16	Mother to Daughter	Father(41); Mother(33)	
DXS7424	18	16-16	18**-18**	Mother to Daughter	Father(30); Mother(22)	
DXS7424	16	15-15	16-**16**	Mother to Daughter	Father(36); Mother(28)	
DXS101	25	24–26	**24–26**	Father to Daughter	Father(35); Mother(37)	0.0011
GATA172D05		8-8	**7**	Mother to Son	Mother(33)	0.0011
GATA165B12	9	10-10	9-9	Mother to Daughter	Father(26); Mother(25)	0.0011
GATA31E08	9	11-11	**9–10**	Mother to Daughter	Father(30); Mother(28)	0.0011
HPRTB	14	12–13	12–15	Father to Daughter	Father(33); Mother(32)	0.0011
DXS8377	45	47-47	**46–**47	Father to Daughter	Father(30); Mother(25)	0.0043
DXS8377		49–53	**50**	Mother to Son	Mother(29)	
DXS8377		46–52	**47**	Mother to Son	Mother(27)	
DXS8377		47–51	**46**	Mother to Son	Mother(33)	

* : In the genotypes of children, alleles with the mutation were denoted in boldface.

## Discussion

### Polymorphism

HWE was performed on female samples, and the genotype distributions did not deviate from HWE at the twenty-six loci. Allele frequencies between female and male samples were not significantly different in all the examined loci. The allele frequencies were 0.0010–0.8164. PIC of all the selected loci reached above 0.59 with the exception of DXS7133, DXS6800 and DXS7423. Power of discrimination in females (PD_F_) was 0.3827–0.9849. Notably, DXS8377, DXS10079, DXS101 and DXS981 are highly polymorphic, with the highest power of discrimination and probability of paternity exclusion among the twenty-six loci studied. These results suggest that the twenty-six X-STR loci are highly polymorphic and have satisfactory forensic efficiency.

### Linkage and linkage disequilibrium

The twenty-six markers reported here were located in four different X-chromosomal linkage groups. DXS6807, DXS8378 and DXS9902 were located in linkage groups 1. The nineteen loci (DXS7132, DXS10079, DXS10074, DXS10075, DXS981, DXS6800, DXS9898, DXS6803, DXS6801, DXS6809, DXS6789, DXS6799, DXS7424, DXS101, DXS7133, DXS6804, GATA172D05, GATA165B12 and DXS6854) were located in linkage groups 2. HPRTB was located in linkage groups 3. GATA31E08, DXS8377 and DXS7423 were located in linkage groups 4. It was found that alleles of linked loci form haplotype that recombine during meioses. When LDE exists, haplotype frequencies have to be estimated directly from appropriate population sample [15]. The two multiplex system may develop haplotypes of the six clusters (cluster I: DXS6807-DXS8378-DXS9902 (Xp22), cluster II: DXS7132-DXS10079-DXS10074- DXS10075-DXS981 (Xq12); cluster III: DXS6801-DXS6809-DXS6789-DXS6799 (Xq21); cluster IV: DXS7424-DXS101-DXS7133 (Xq22), cluster V: DXS6804-GATA172D05 (Xq23), cluster VI: DXS8377-DXS7423 (Xq28)). A total of 89, 703, 335, 147, 39 and 63 haplotypes were observed and haplotype diversity was 0.9584, 0.9994, 0.9935, 0.9736, 0.9427 and 0.9571 for cluster I, II, III, IV, V and VI, respectively. The Uigur population showed the highest level of LDE. In this population, significant LDE (*P*<0.00001) was observed in cluster II and III. The *P* value of the exact test for LDE is different in different populations. It is possible that this association was the result of sample size.

### Comparisons among different populations

The comparisons of the allele frequency distribution were performed among our studied populations as well as between our selected populations and those reported by others, such as Sichuan Han [1], Taiwan [3], Japan [4], Pakistan [16], Northern Italy [17], Brazil [18], Algeria [19], Ghana [20], and Ivory Coast [21]. Significant differences were found in the selected 21 loci between Han and Uigur, in the selected 24 loci between Han and Kazakh, and in the selected 16 loci between Han and Mongol. However, no significant differences were found between Guangdong Han and Sichuan Han as well as Taiwanese Han. Probably this is because most Taiwanese come from Han population living in Mainland China. Significant differences were found between Uigur and Mongol in the selected 13 loci, but no significant differences were found between Uigur and Kazakh in the selected 20 loci. Heterogeneous marriage or marriage between different regions is not common and homogeneous marriage or marriage within the same region is prevalent because of differences in nationality origin, language and culture, etc. The Uigur are originated from ancient HuiGe. The Kazakh are originated in the central Asian steppes. In the middle of the sixth century, Kazakh and Uigur were affected by the Turkish culture. There are many similarities between Uigur, Kazakh, and Turkish ethnic languages and cultures. So intermarriage among the Uigur, kazakh and Turkish is common. This may possibly explain why there is no significant difference between the Uigur and the Kazakh. Moreover, there are significant differences of haplotype distribution in the five clusters between the Uigur and the Kazakh except at the clusters VI (DXS8377/DXS7423). Notably, the same haplotype in clusters II (DXS7132-DXS10079-DXS10074-DXS10075-DXS981) has only nine between the Uigur and the Kazakh. Significant differences were found between Kazakh and Mongol in the selected 10 loci. Besides, significant differences were also found in a great number of loci between our selected populations and those of other countries (Table S2). As a result, allele frequency distribution for most X-STR loci is different in different populations. So it is important to develop population data for forensic analysis.

### Mutation

In the kinship cases, 40 three-generation families (grandmother-father- granddaughter) have been tested using MX15-STR and MX12-STR. The grand-maternal genotypes were found to be transmitted to her granddaughters by her son. Thirty-one mutations were detected from the twenty-six loci in 24,336 meioses. The average mutation rate for the twenty-six loci was estimated to be 1.27×10^−3^ per meiosis. 96.77% mutation is the shift of one repeat unit. Our results are consistent with those of Fracasso [22], Shin [23] and Szibor et al [24]. Mutation rate of the same order was also described for autosomal STR [25].

## Conclusion

Our results suggest that allele frequency distribution for most X-STR loci is population-specific and the haplotypes of the six clusters may provide a powerful tool for haplotype analysis in kinship testing and relationship identification. So it is necessary to acquire allele frequency and haplotypes data of the linked loci in different ethnic groups for forensic application.

## Supporting Information

File S1 **Sequencies of some alleles for 26 X-STR loci.**
(PDF)

Table S1 **p -value for allele frequency distribution of 26 X-STR loci among the selected four nationality data.**
(XLS)

Table S2 **p-value for allele frequency distribution between the four selected population and previously published population data.**
(XLS)

Table S3 **Haplotype of DXS6807-DXS8378-DXS9902.**
(XLS)

Table S4 **Haplotype of DXS7132-DXS10079-DXS10074-DXS10075-DXS981.**
(XLS)

Table S5 **Haplotype of DXS6801-DXS6809-DXS6789-DXS6799.**
(XLS)

Table S6 **Haplotype of DXS7424-DXS101-DXS7133.**
(XLS)

Table S7 **Haplotype of DXS6804-GATA172D05.**
(XLS)

Table S8 **Haplotype of DXS8377-DXS7423.**
(XLS)
