# Supplementary material for: Allele and Haplotype Diversity of 26 X-STR Loci in Four Nationality Populations from China
Source: PLoS One. 2013 Jun 21;8(6):e65570. doi: 10.1371/journal.pone.0065570 (PMC3689794; doi:10.1371/journal.pone.0065570)
Supplement: File S1 — Sequencies of some alleles for 26 X-STR loci. (PDF) [file pone.0065570.s001.pdf]

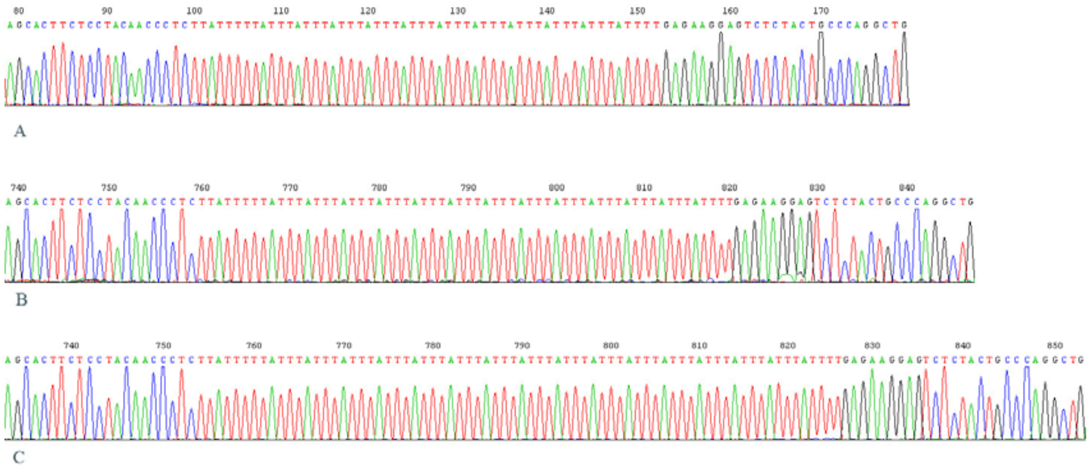

Fig S1. Sequences of allele 11, 13 and 16 at the locus DXS6854 (A: 11 ； B:13；C： 16)

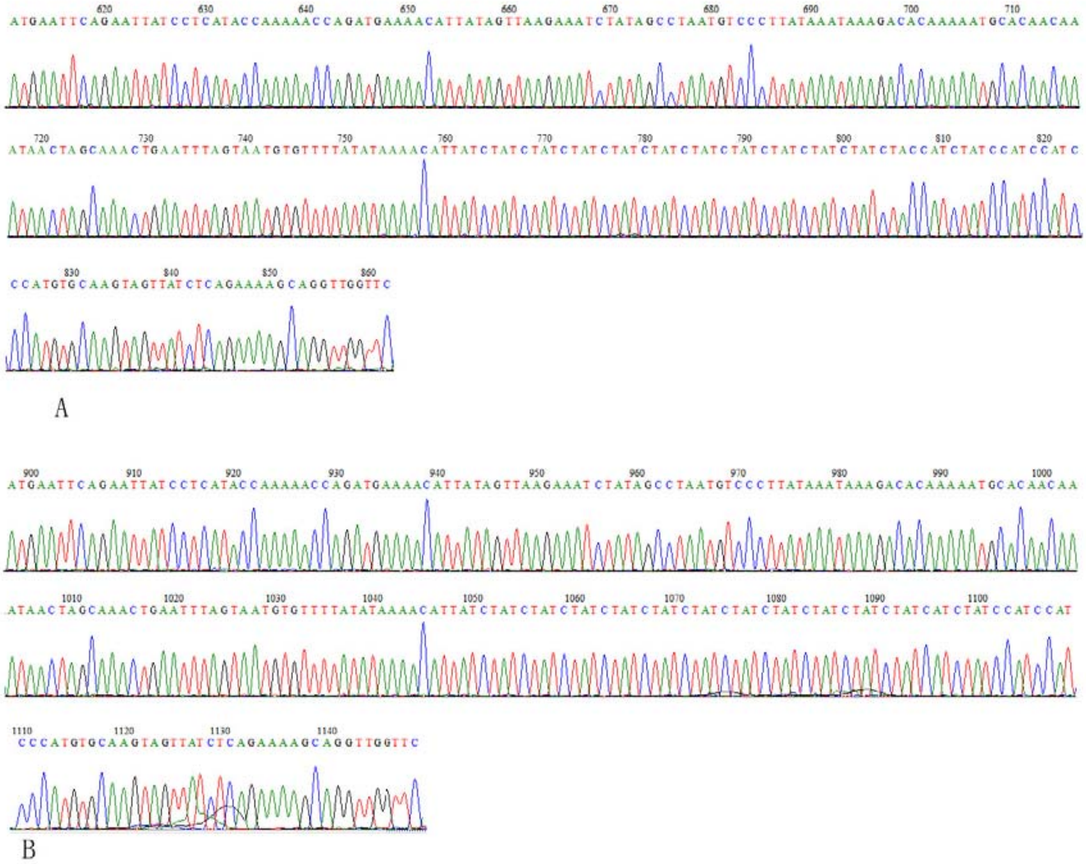

Fig S2. Sequences of allele 12 at the locus DXS6799

A：12 P_{F25}-N_{125}-$[TATC]_{11}$ –TACC-N_{34}-P_{R20}

B：12a P_{F25}-N_{125}-$[TATC]_{12}$ -N_{34}-P_{R20}

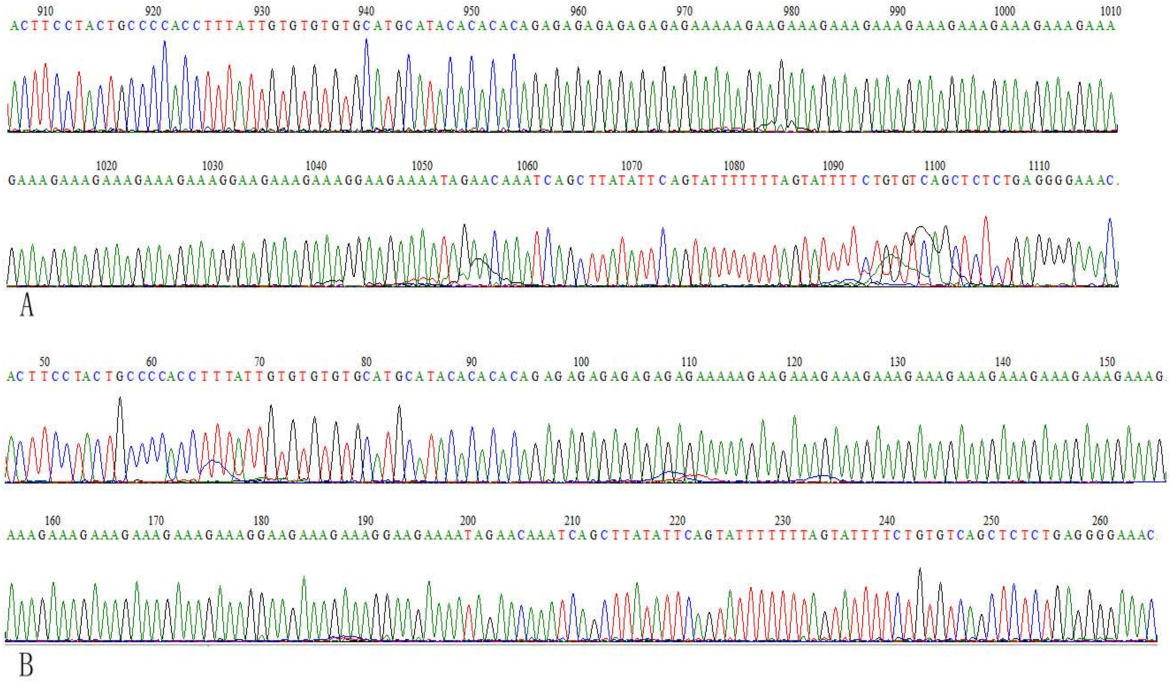

Fig S3. Sequences of allele 16 and 18 at the locus DXS10074

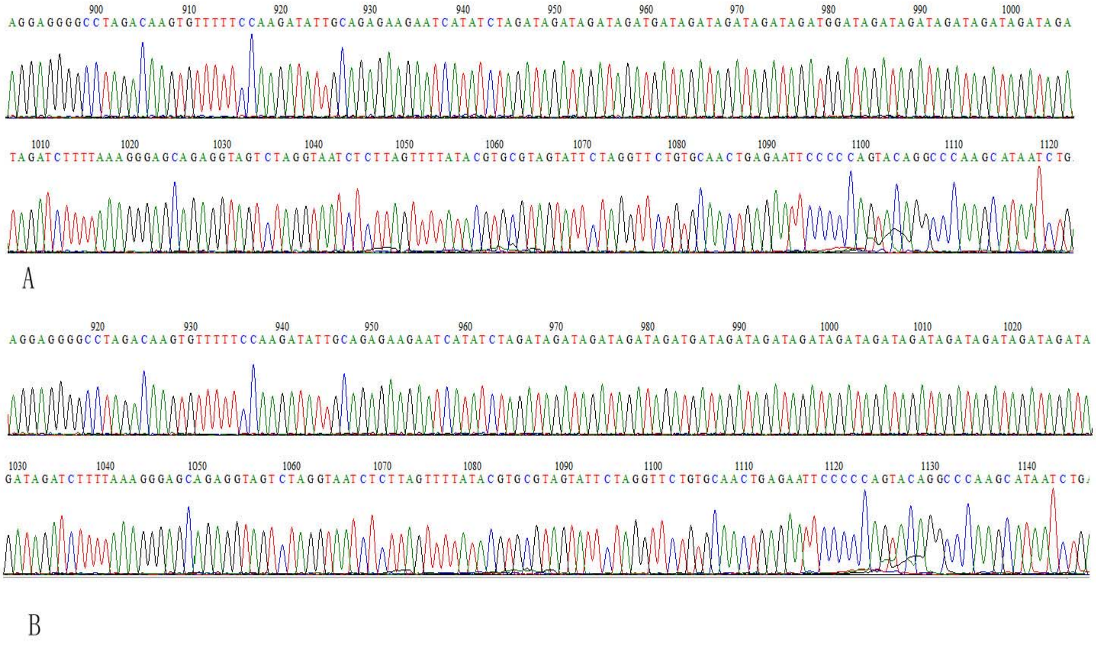

Fig S4. Sequences of allele 16 and 17 at the locus DXS10074（A：16；B：17）

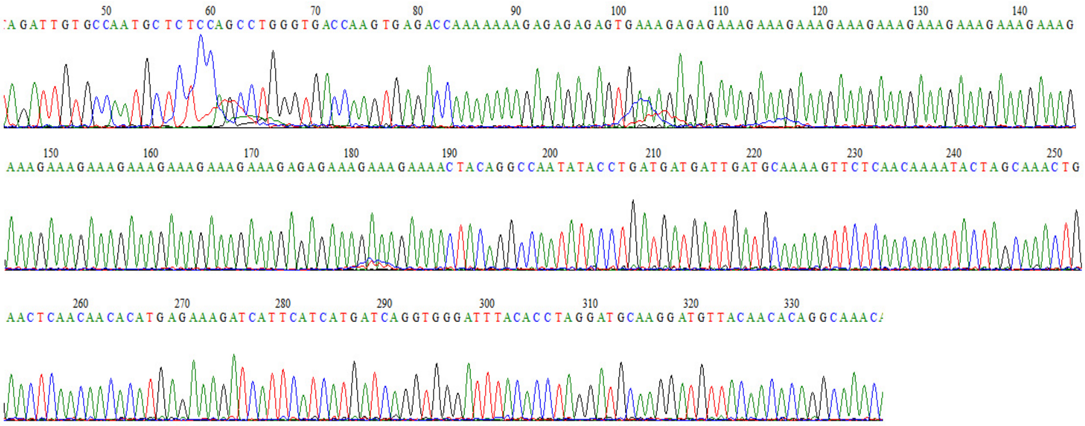

FigS5. Sequences of allele 20 at the locus DXS10079

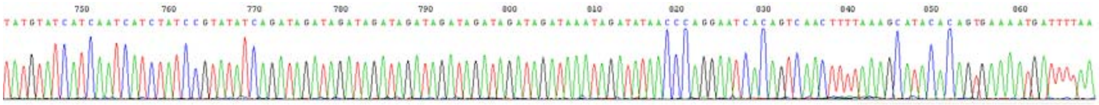

Fig S6. Sequences of allele 9 at the locus GATA165B12

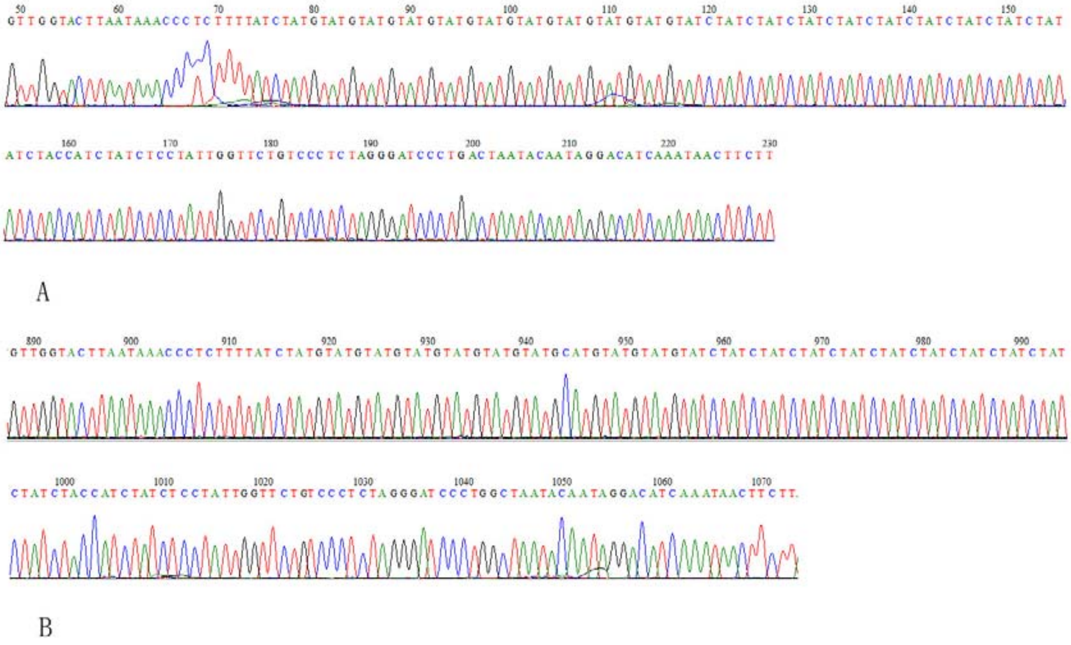

Fig S7. Sequences of allele 21and 22 at the locus DXS6789（A：21；B：22）

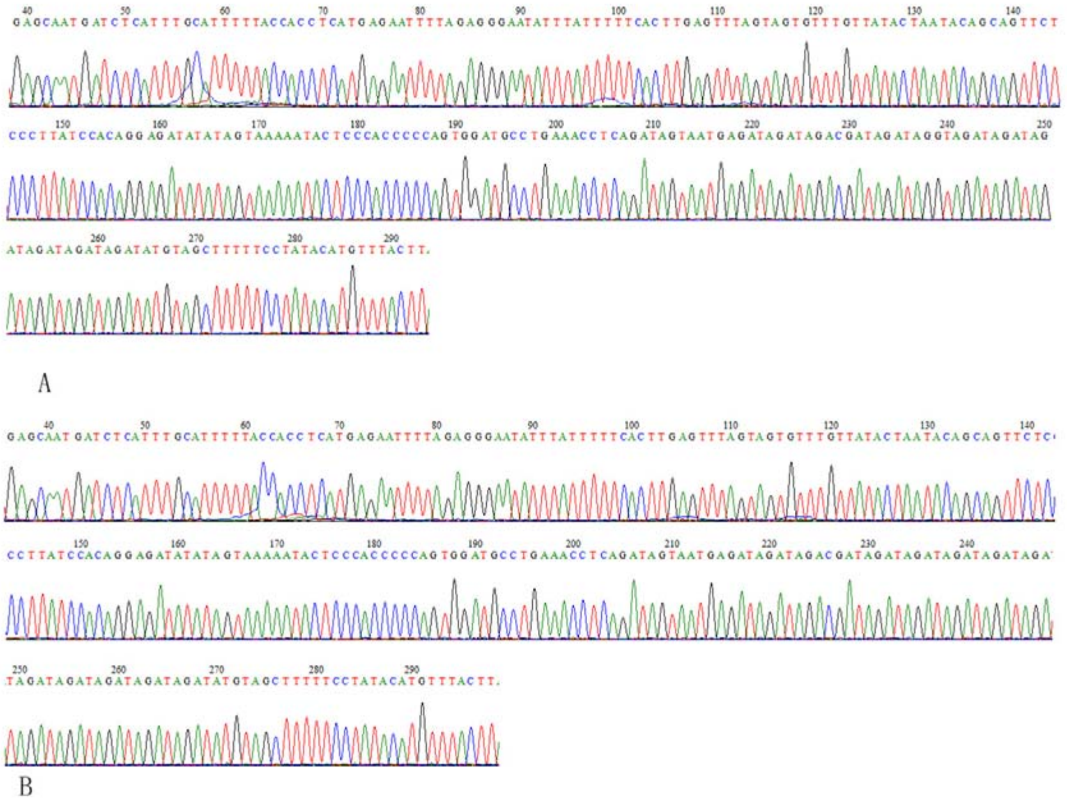

Fig S8. Sequences of allele 12 and 14 at the locus DXS6807（A：12；B：14）

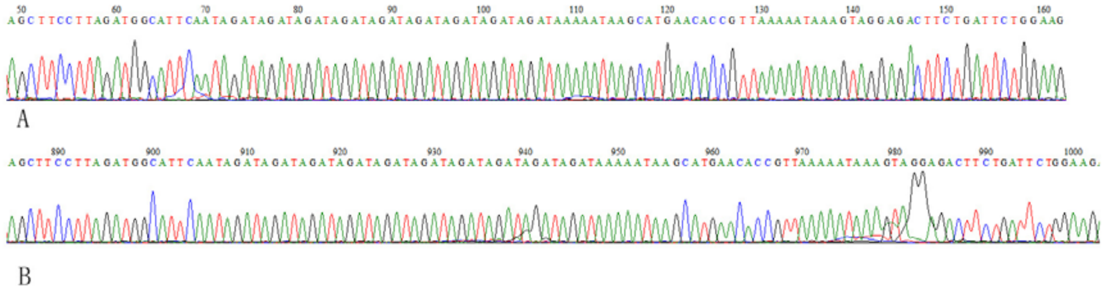

FigS9. Sequences of allele 9 and 10 at the locus DXS7133（A：9；B：10）

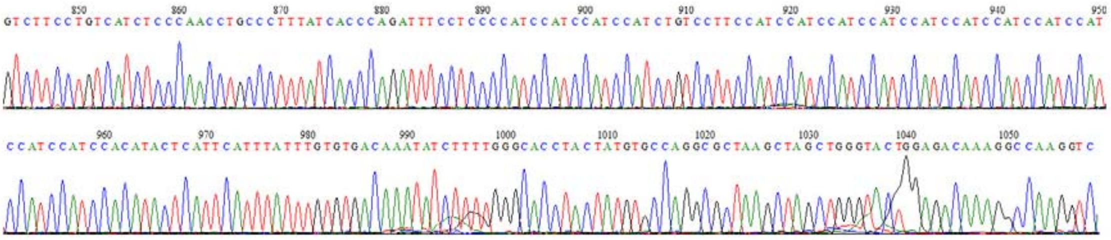

Fig S10. Sequences of allele 15 at the locus DXS7423

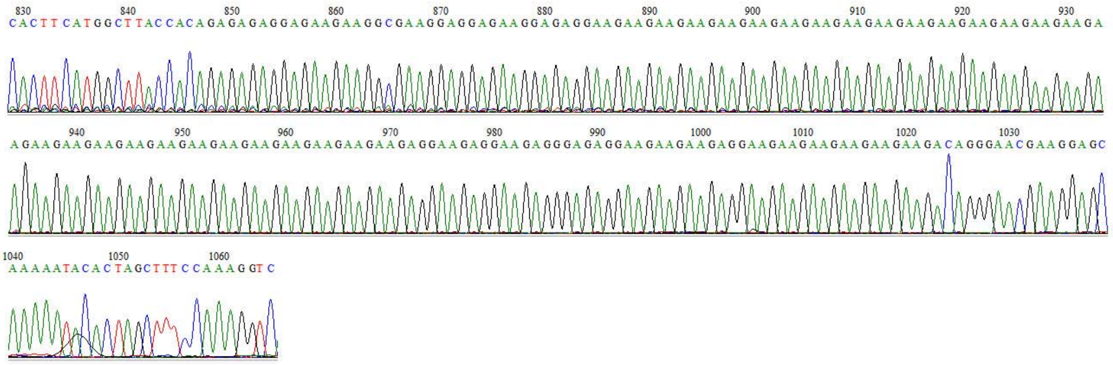

Fig S11. Sequences of allele 47 at the locus DXS8377

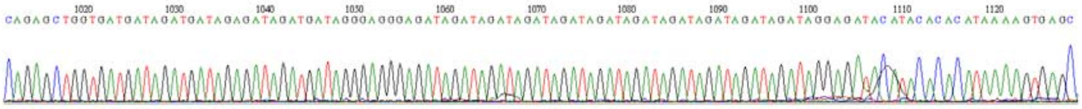

Fig S12. Sequences of allele 11 at the locus GATA31E08

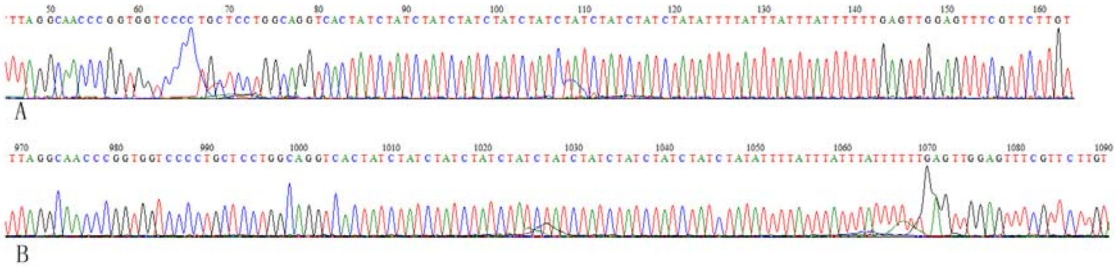

Fig S13. Sequences of allele 10 and 11 at the locus DXS8378（A：10；B：11）

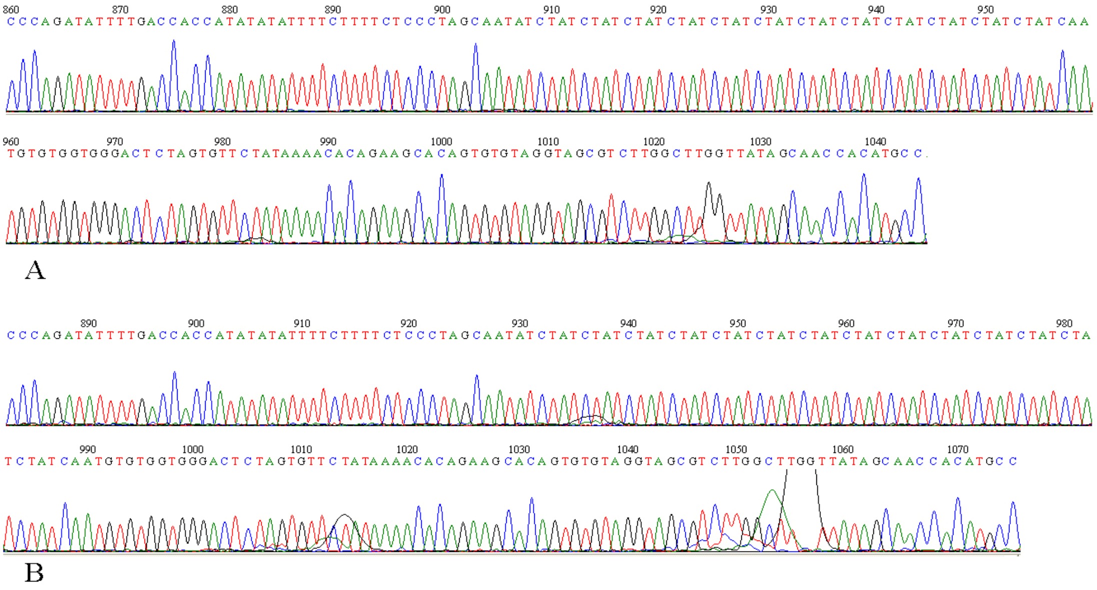

Fig S14. Sequences of allele 13 and 15 at the locus DXS6804 for 9947A sample（A：13；B：15）

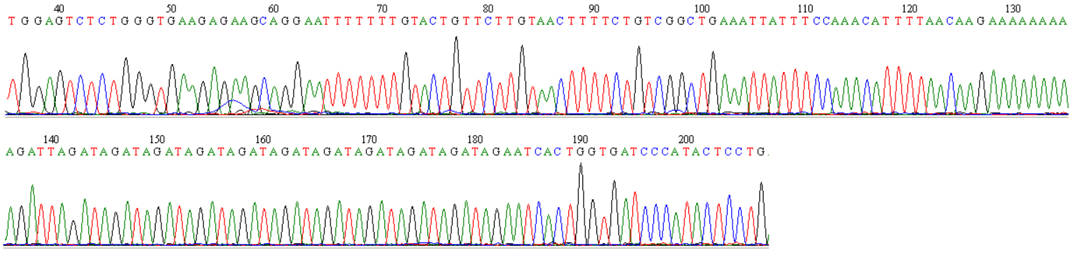

Fig S15. Sequences of allele 11 at the locus DXS9902 for 9947A sample

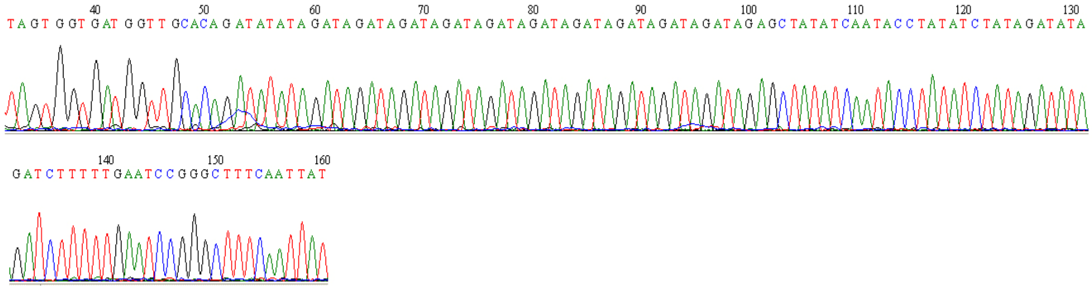

Fig S16. Sequences of allele 11 at the locus GATA172D05 for 9947A sample

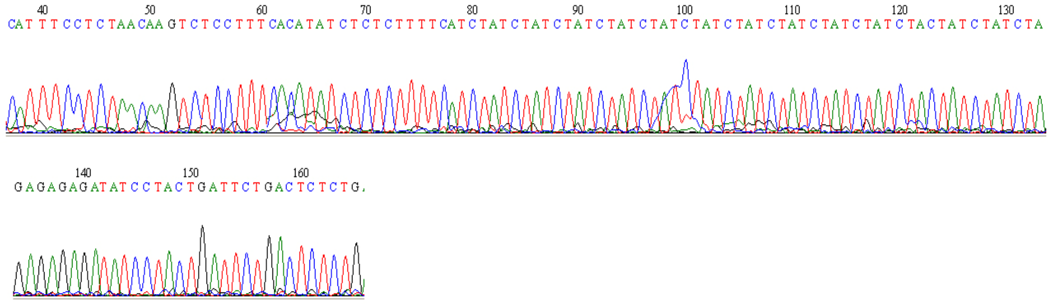

Fig S17. Sequences of allele 13 at the locus DXS6801

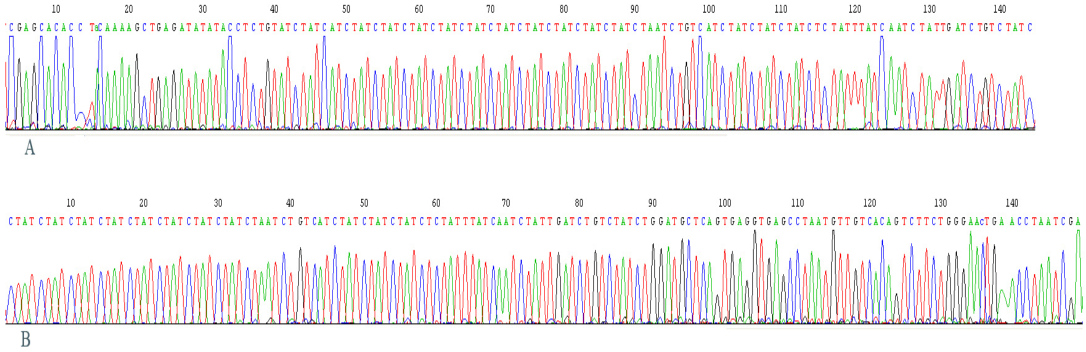

FigS18. Sequences of allele 12 at the locus DXS9898 (A: reverse ； B: forward)

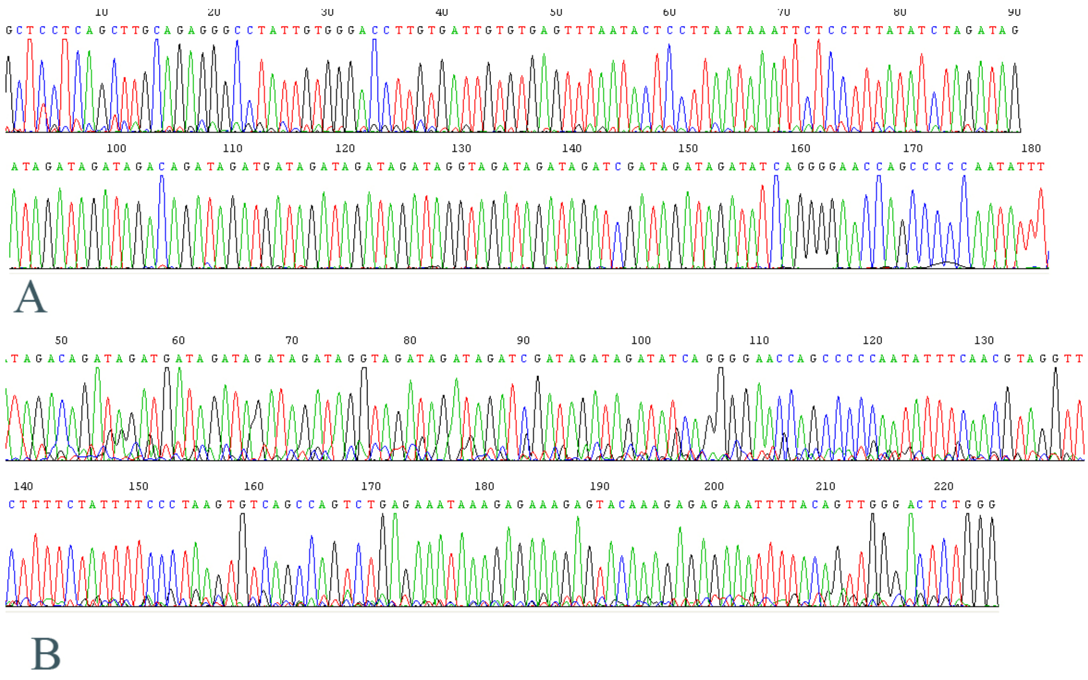

Fig S19. Sequences of allele 16 at the locus DXS6800 (A: reverse, B: forward)

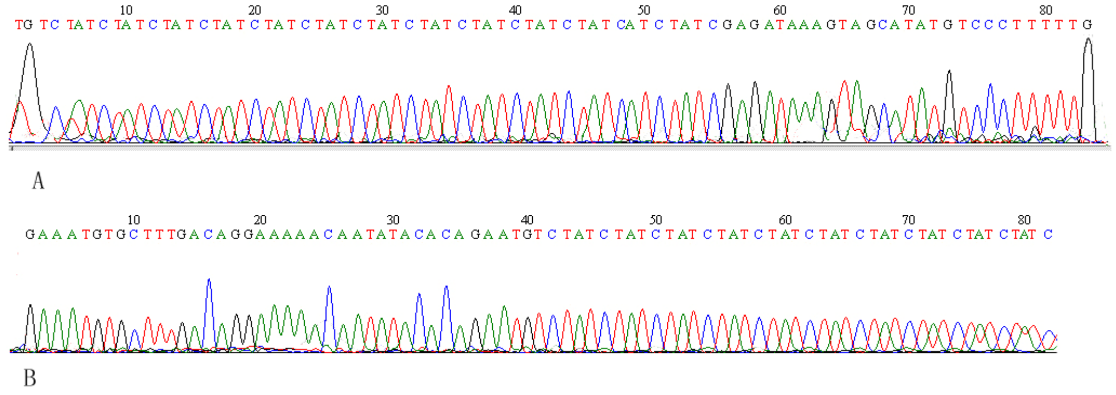

Fig S20. Sequences of allele 12.3 at the locus DXS6803 (A: forward, B: reverse)

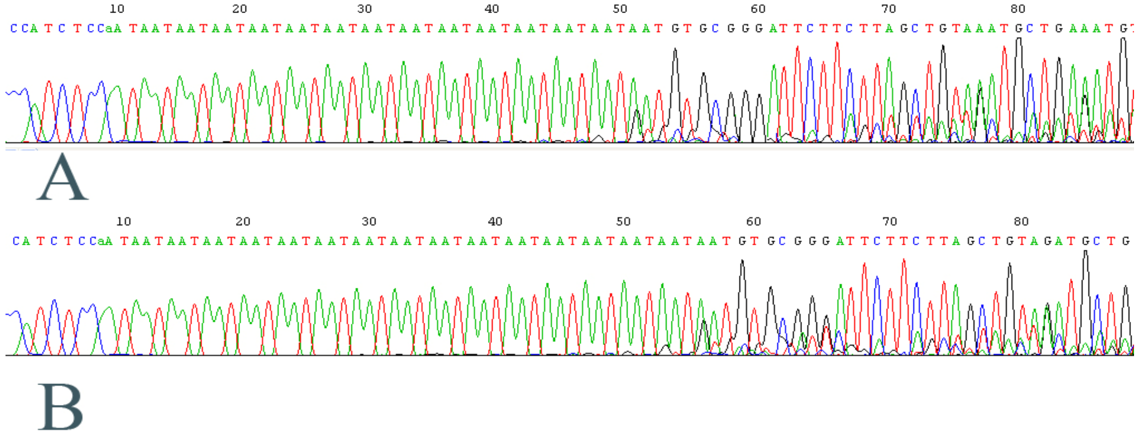

Fig S21. Sequences of allele 14 and 16 at the locus DXS7424 for 9947A sample （A：14；B：16）

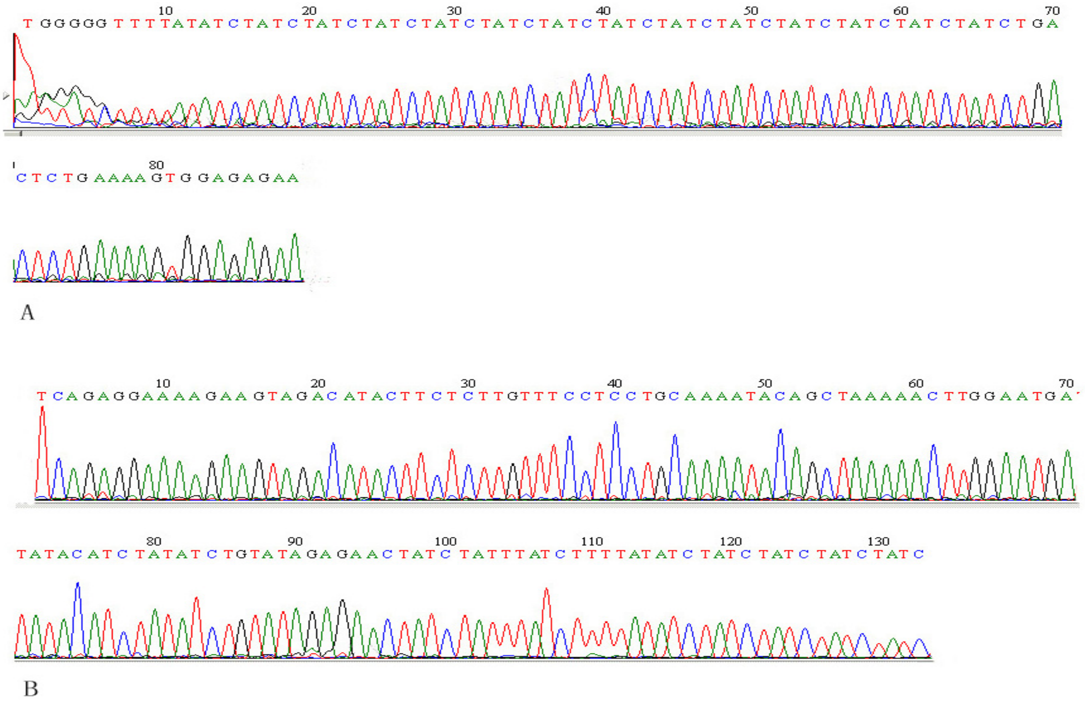

Fig S22. Sequences of allele 14 at DXS981 (A: forward, B: reverse)

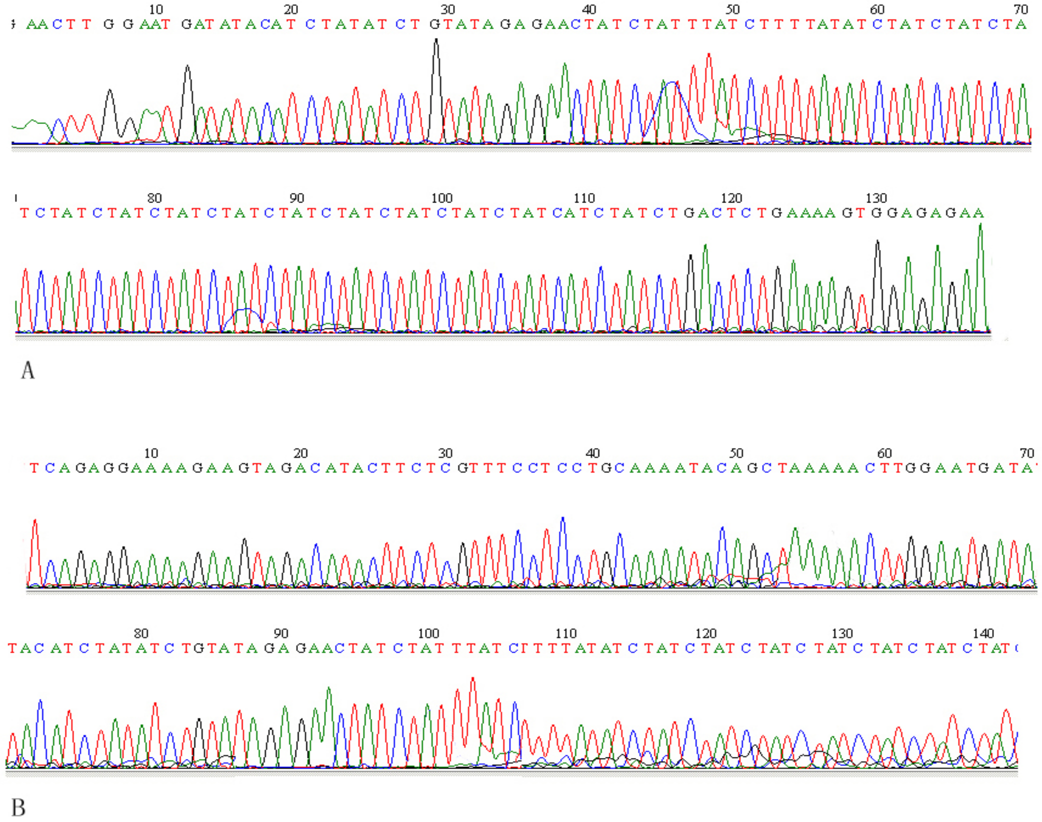

Fig S23. Sequences of allele 14.3 at DXS981 (A: forward, B: reverse)

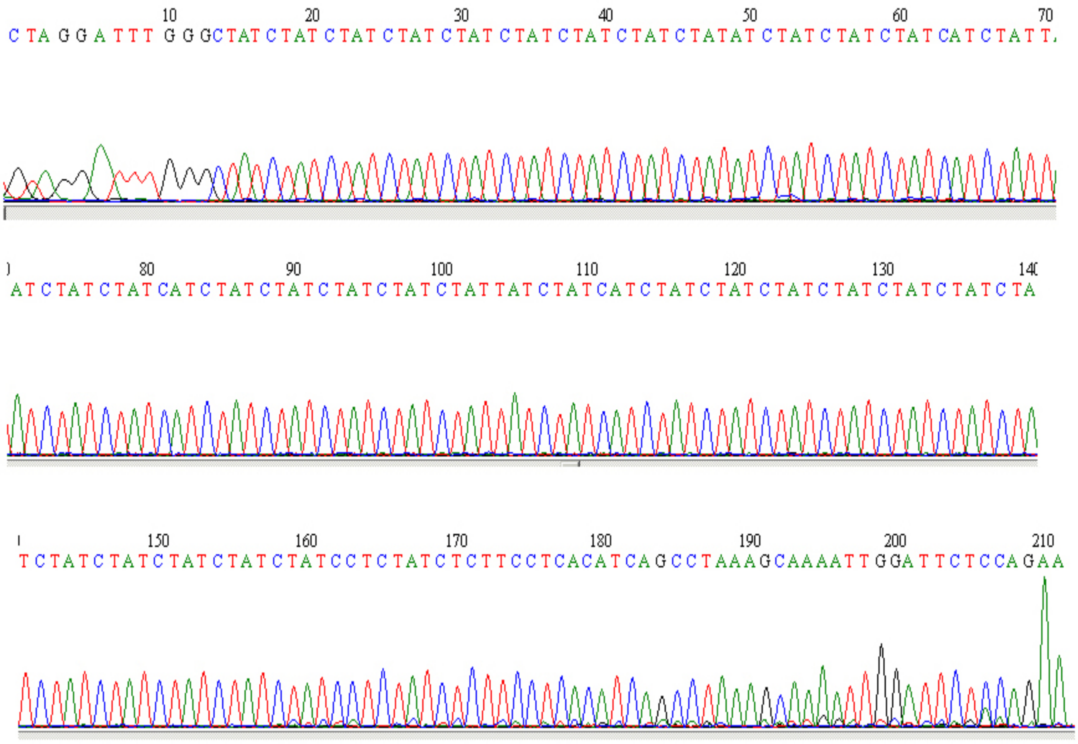

A

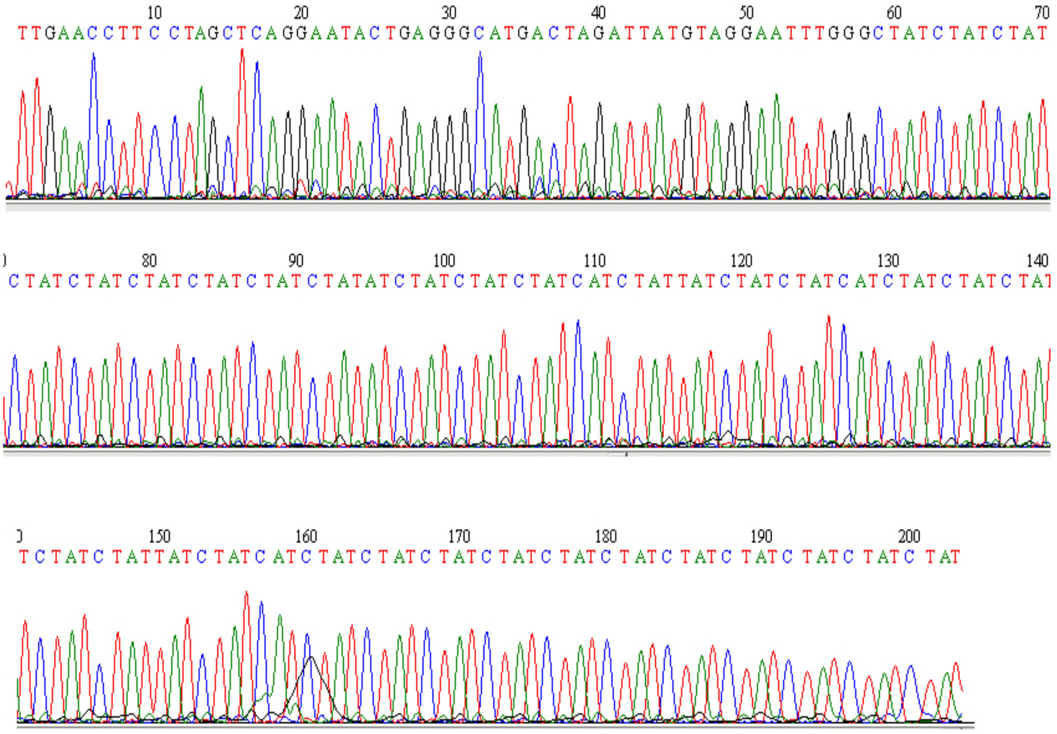

B

Fig S24. Sequences of allele 32 at the locus DXS6809 (A: forward, B: reverse)

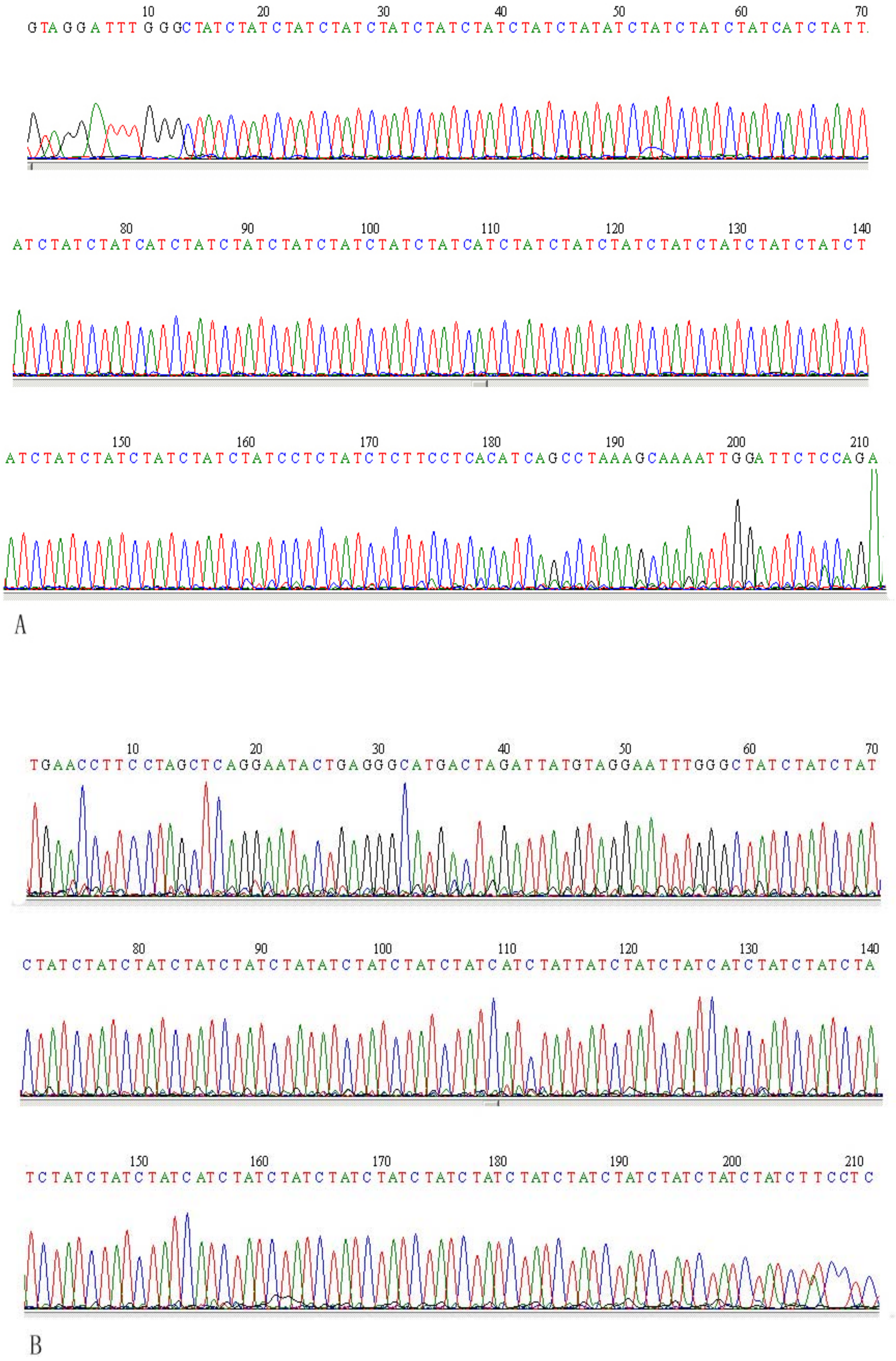

Fig S25. Sequences of allele 32.1 at the locus DXS6809 (A: forward, B: reverse)

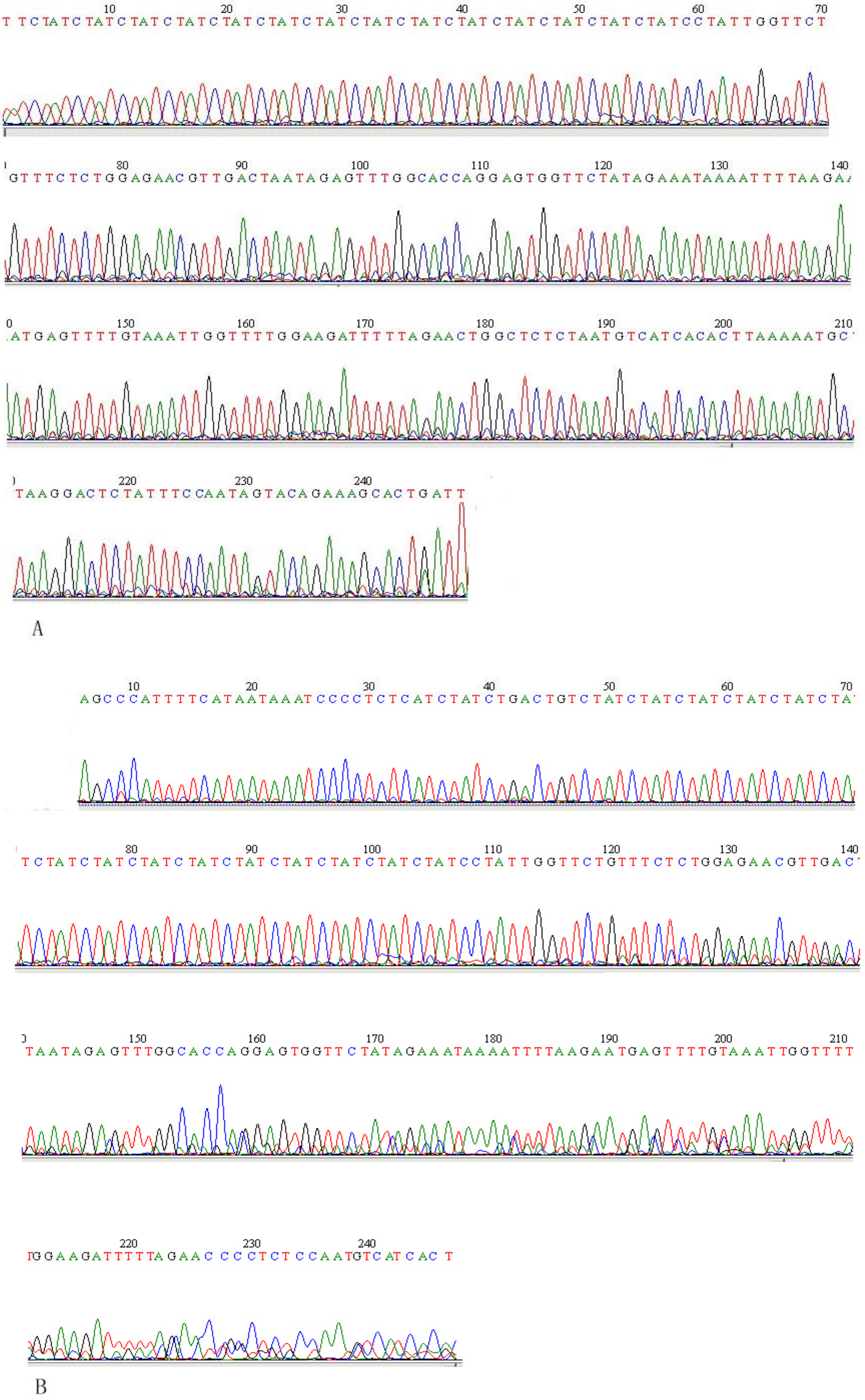

Fig S26. Sequences of allele 15 at the locus DXS7132 (A: forward, B: reverse)

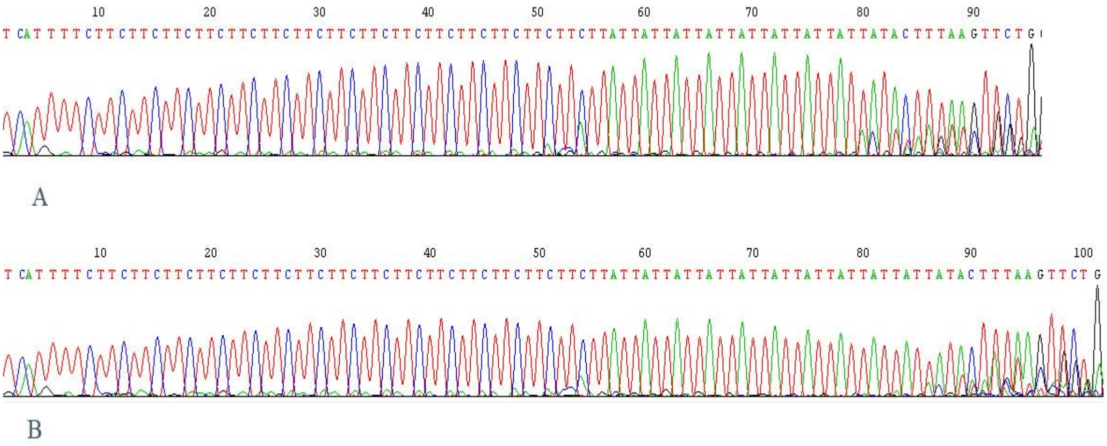

FigS27. Sequences of allele 24 and 26 at the locus DXS101 for 9947A sample （A：24；B：26）

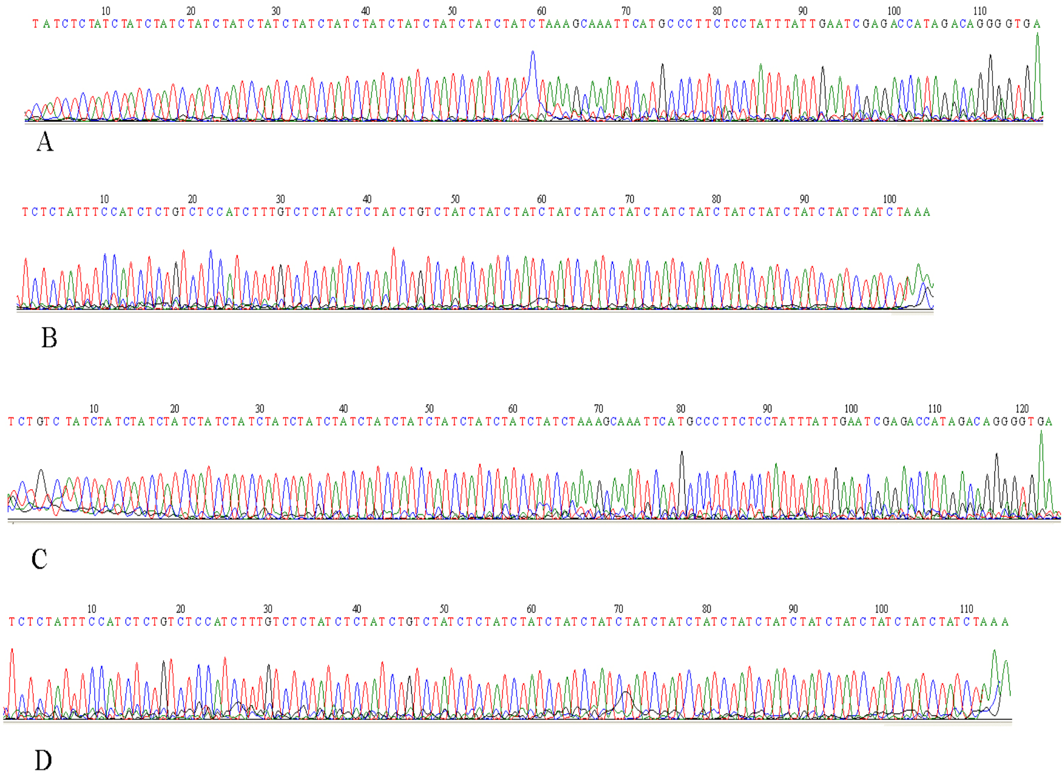

Fig S28. Sequences of allele 13、13.2 and 14 at the locus HPRTB

(A: 13 forward, B: 13.2 reverse, C：13.2 forward, D：14 reverse)
